# Uniaxial stress control of skyrmion phase

**DOI:** 10.1038/ncomms9539

**Published:** 2015-10-13

**Authors:** Y. Nii, T. Nakajima, A. Kikkawa, Y. Yamasaki, K. Ohishi, J. Suzuki, Y. Taguchi, T. Arima, Y. Tokura, Y. Iwasa

**Affiliations:** 1 RIKEN Center for Emergent Matter Science (CEMS), Wako 351-0198, Japan; 2 Department of Applied Physics and Quantum-Phase Electronics Center (QPEC), University of Tokyo, Tokyo 113-8656, Japan; 3 Research Center for Neutron Science and Technology, Comprehensive Research Organization for Science and Society (CROSS), Tokai, Ibaraki 319-1106, Japan; 4 Department of Advanced Materials Science, University of Tokyo, Kashiwa 277-8561, Japan

## Abstract

Magnetic skyrmions, swirling nanometric spin textures, have been attracting increasing attention by virtue of their potential applications for future memory technology and their emergent electromagnetism. Despite a variety of theoretical proposals oriented towards skyrmion-based electronics (that is, skyrmionics), few experiments have succeeded in creating, deleting and transferring skyrmions, and the manipulation methodologies have thus far remained limited to electric, magnetic and thermal stimuli. Here, we demonstrate a new approach for skyrmion phase control based on a mechanical stress. By continuously scanning uniaxial stress at low temperatures, we can create and annihilate a skyrmion crystal in a prototypical chiral magnet MnSi. The critical stress is merely several tens of MPa, which is easily accessible using the tip of a conventional cantilever. The present results offer a new guideline even for single skyrmion control that requires neither electric nor magnetic biases and consumes extremely little energy.

## Abstract (web summary)

Chiral magnets can support particle-like magnetization textures called skyrmions which form in lattices and can be manipulated for potential device applications. Here, the authors demonstrate the controlled creation and annihilation of a skyrmion lattice in MnSi single crystals using mechanical stress.

## Graphical abstract

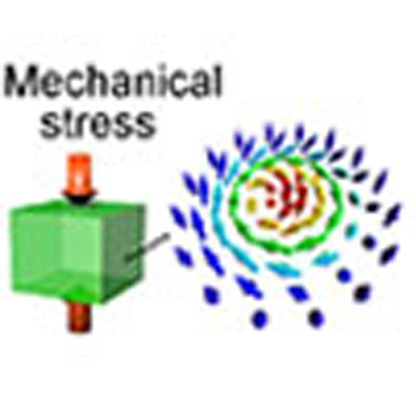

Magnetic skyrmions and their crystalline form called skyrmion crystals (SkX; Fig. 1a,b), have been observed in bulk materials^1^,^2^,^3^,^4^, thin films^5^,^6^,^7^ and nanowires^8^,^9^ of *P*2_1_3-type chiral magnets such as MnSi, Fe_1−*x*_Co_*x*_Si and Cu_2_OSeO_3_. They have also been observed quite recently in non-*P*2_1_3-type chiral magnets of GaV_4_S_8_ (ref. 10) and Co-Zn-Mn alloys^11^, and even at the interfaces of magnetic thin films^12^,^13^ and nanoscopic-patterned discs^14^,^15^. As schematically illustrated in Fig. 1a, a skrymion has a particle-like swirling spin configuration. Unlike the conventional spin textures such as helical or conical states (Fig. 1c) that appear in chiral magnets, skyrmions have spin configurations characterized by a topological index called the skyrmion number^16^,^17^. The nontrivial topology of skyrmions gives rise to emergent electromagnetic responses as exemplified by the topological Hall effect^18^, skyrmion magnetic resonance^19^ and thermally induced ratchet motion^20^.

In addition to these intriguing physical responses, skyrmions have been attracting extensive attention for their potential applications in future solid-state memory technology or storage devices^21^,^22^ because of their small size (3–100 nm)^21^ and topological stability^16^ as well as ultralow electric currents required to drive their motion^23^,^24^,^25^. Pursuing arbitrary control of skyrmions as information carriers towards the attempt to develop skyrmion-based electronics (that is, skyrmionics), a number of theoretical simulations have recently been performed, yielding schemes for controlling skyrmions based on electric currents^22^,^26^,^27^,^28^,^29^,^30^,^31^,^32^,^33^, electric fields^32^,^34^, magnetic fields^32^, thermal stimuli^35^ or conversions between a skyrmion and domain walls^36^. Compared with these theoretical simulations, experimental demonstrations such as the creation^13^,^37^, deletion^13^, transfer^23^,^24^,^25^ and rotation^20^,^38^ of skyrmions seem to be limited. Therefore, it remains necessary to establish and expand a framework for skyrmion manipulation.

Herein, we present a new approach for manipulating a SkX in a bulk single crystal of a prototypical chiral magnet MnSi. Although any continuous deformations of the spins cannot connect topologically different skyrmion and conical states, we find that a simple mechanical stress can overcome the energy barrier to switch between these two states in both directions. The critical stress needed to trigger this topological switching is merely several tens of MPa, and the corresponding strain is of the order of 10^−4^. This perturbative mechanical stimulus is easily achievable using conventional scanning probe techniques, offering a new guideline for skyrmion manipulation, even at the single skyrmion level, that is essentially free of Joule heating.

## Results

### Experimental set-up for the application of mechanical stress

**Figure 1:**
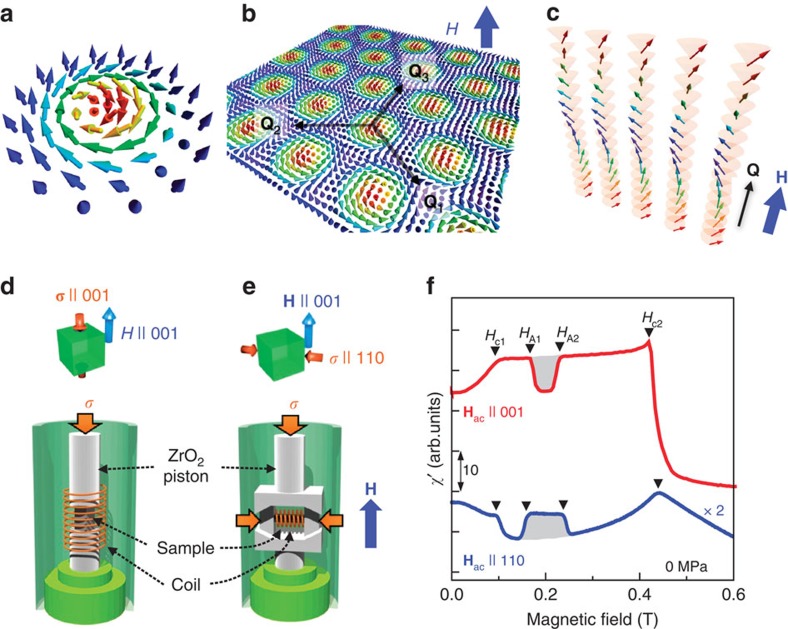
Conceptual representations of magnetic phases in MnSi and the experimental set-up. (**a**) Single skyrmion, (**b**) skyrmion crystal and (**c**) conical phase. **H** and **Q**_*i*_ represent the magnetic field and propagation vectors, respectively. The colours of the arrows indicate the out-of-plane components of the spins in the skyrmion spin texture, whereas they represent the directions of the in-plane components (perpendicular to the Q-vector) in the conical structure. (**d**,**e**) The set-up for AC magnetic susceptibility measurements under (**d**) longitudinal and (**e**) transverse uniaxial stresses with respect to an external magnetic field. The (green) cuboids represent a cubic unit cell, and **σ** represents the compressive stress. In the transverse configuration, several ZrO_2_ pistons converted longitudinal forces into lateral ones. The magnetic susceptibility was monitored by coils. (**f**) Typical results for *χ′* as a function of the magnetic field at 0 MPa. The red and blue curves correspond to the longitudinal and transverse AC magnetic susceptibilities, which were measured in configurations (**d**,**e**), respectively. These data are shown separately by an offset. Alternating magnetic fields (**H**_ac_) were applied by a coil parallel to the **σ** axis. The critical fields corresponding to the phase transitions from the helical phase to the conical phase and from the SkX phase to the induced ferromagnetic phase are represented by *H*_c1_ and *H*_c2_, respectively. The SkX phase can be clearly assigned, as represented by the shaded regions between *H*_A1_ and *H*_A2_.

We continuously applied and released compressive uniaxial stress over broad ranges of temperature and magnetic field strengths using a previously developed special uniaxial stress probe^39^ that was improved for the present study. As shown in Fig. 1d,e, longitudinal (**σ** || **H**) or transverse (**σ** ⊥ **H**) uniaxial stress was introduced, and the longitudinal or transverse AC magnetic susceptibility was simultaneously monitored by coils. Here, **σ** and **H** represent the compressive stress and magnetic field, respectively. In both configurations, a DC magnetic field was applied in the 001 direction, and the uniaxial stress and AC magnetic field were set parallel to each other.

Figure 1f presents a typical evolution of the AC magnetic susceptibility of a MnSi single crystal under a magnetic field sweep at 0 MPa for these two configurations. The red and blue curves correspond to the longitudinal and transverse AC magnetic susceptibilities, respectively. As the magnetic field in 001 direction increases, a transition from the multi-domain helical phase to the single-domain conical phase occurs at *H*_c1_, followed by another transition from the conical phase to the induced ferromagnetic phase at *H*_c2_. In the intermediate range between *H*_A1_ and *H*_A2_, as represented by the grey regions, the longitudinal susceptibility shows a clear suppression, whereas the transverse susceptibility exhibits a clear enhancement. This behaviour is ascribable to the SkX phase. In the case of proper helices, the susceptibility parallel to the propagation vector **Q** is expected to be larger than that perpendicular to the **Q**-vector. The **Q** direction of the conical phase is parallel to the **H** direction, whereas that of the SkX phase is orthogonal (Fig. 1b,c). This difference in **Q**-vector direction qualitatively explains the opposite trends exhibited by the longitudinal and transverse susceptibilities in the SkX and conical phases. The slight discrepancies between the critical fields for the two configurations are attributed to the demagnetizing effect.

### Mechanical creation and annihilation of SkX

**Figure 2:**
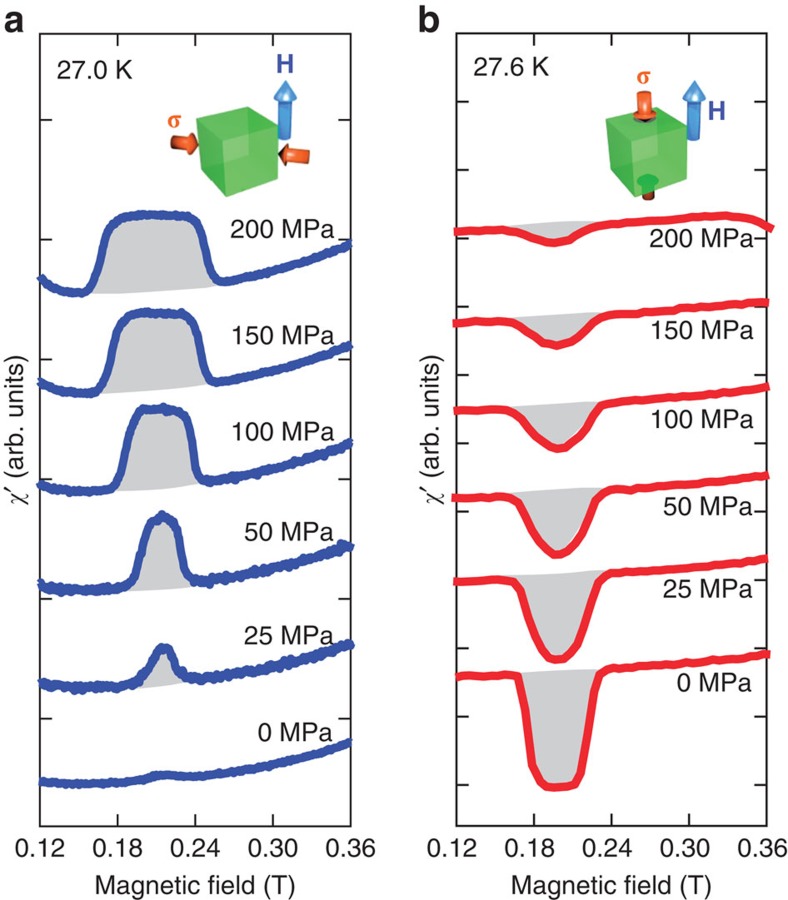
Mechanical creation and annihilation of SkX. (**a**) Emergence of the SkX phase at 27.0 K. With increasing transverse stress, as schematically shown in the inset, the magnetic susceptibility near ∼0.2 T begins to increase, as indicated by the grey-shaded region. The appearance of a convex structure in the transverse magnetic susceptibility indicates the emergence of the SkX phase. (**b**) Annihilation of the SkX phase at 27.6 K. With increasing longitudinal stress, the concave structure (shaded in grey) in the longitudinal magnetic susceptibility nearly disappears. This indicates suppression of the SkX phase. Both sets of data are shown separately by offsets.

We measured the uniaxial stress dependence of the magnetic susceptibilities at low temperatures. Figure 2a,b present the AC susceptibilities near 0.2 T with increasing mechanical stresses, which unambiguously demonstrated that the SkX phase is created by a transverse stress and annihilated by a longitudinal stress. At 27.0 K in the absence of stress, there is no SkX phase. On the application of a stress in the transverse configuration (**σ** ⊥ **H**), as shown in the inset of Fig. 2(a), *χ′* begins to increase at ∼0.2 T, indicating the emergence of the SkX phase. At 27.6 K, however, the SkX phase exists at 0 MPa, as indicated by a reduction in *χ′* (Fig. 2b) in the longitudinal configuration (**σ** || **H**). This reduction is continuously suppressed with increasing uniaxial stress up to 200 MPa, indicating that the SkX phase becomes unstable on the application of longitudinal uniaxial stress and that the conical phase survives between *H*_A1_ and *H*_A2_. These results demonstrate that even moderate longitudinal or transverse stresses of up to 200 MPa are sufficient to control the competition between the SkX and conical phases.

**Figure 3:**
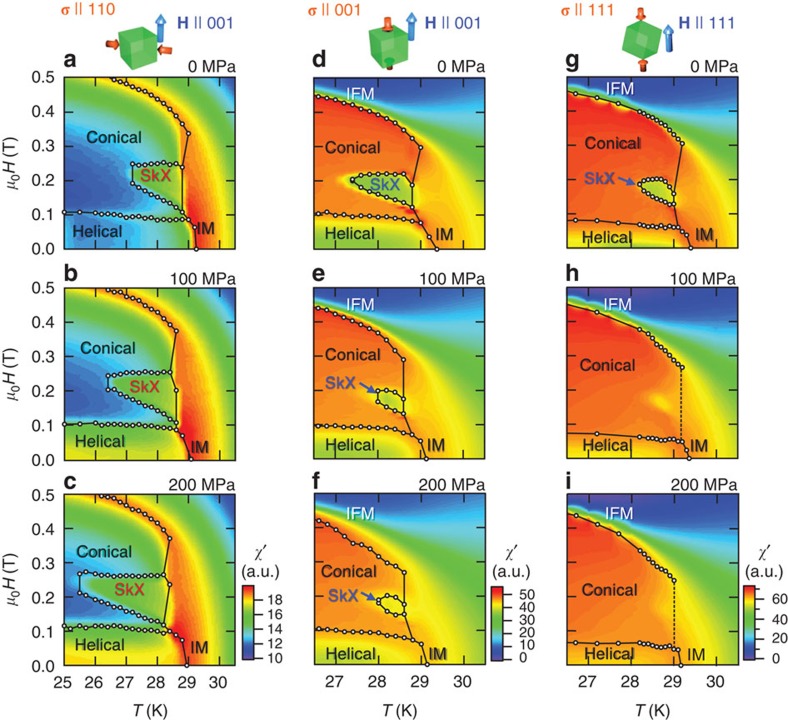
Evolution of the magnetic phase diagram under mechanical stress at 0, 100 and 200 MPa. The relative stability of the SkX phase can be controlled by applying a transverse or longitudinal stress along different crystallographic axes, as schematically illustrated here. (**a**–**c**) Expansion of the SkX phase region induced by a transverse stress along the 110 axis with *H* || 001. (**d**–**f**) Shrinkage of the SkX phase region induced by a longitudinal stress along the 001 axis with *H* || 001. (**g**–**i**) Collapse of the SkX phase induced by a longitudinal stress along the 111 axis with *H* || 111. The colour contour maps correspond to the *χ′* values obtained in a series of *H*-increasing scans at fixed temperatures and stresses. The demagnetization effects were not corrected. IFM and IM indicate the induced ferromagnetic and intermediate phases, respectively. The dotted lines in (**h**) and (**i**) are guides for the eye.

Figure 3 exhibits an evolution of the magnetic phase diagram under several fixed uniaxial stresses up to 200 MPa in the transverse and longitudinal configurations. The magnetic phase diagrams shown in Fig. 3a–f were obtained in the same settings represented in Fig. 1d,e, respectively. Note that Fig. 3g–i were obtained in a **H** || **σ** || 111 configuration; this is the only instance of data collected in this different setting in the manuscript. At 0 MPa, the SkX phase exists in a small *H*–*T* region surrounded by the conical and intermediate^40^ phases. With a magnetic field along the 111 direction, the SkX phase exists in a narrower region than it does in the case of a magnetic field along the 001 direction. This anisotropy is consistent with a previous report^41^. The slight discrepancy between Fig. 3a,d originates from the demagnetization effect, which depends on the sample shape used in the uniaxial stress experiments (see Methods). The relative stability of the SkX phase can be varied by applying a transverse or longitudinal stress: the SkX phase extends to lower temperatures on the application of a transverse stress (Fig. 3a–c), whereas its temperature range narrows on the application of a longitudinal stress (Fig. 3d–f,g–i). Although the collapse of the SkX phase is incomplete even at 200 MPa with the application of stress along the 001 axis, the SkX phase disappears throughout the entire *H*–*T* region when a longitudinal stress is applied along a magnetically hard axis, that is, the 111 axis (Fig. 3i). A large response also arises in the helical phase in both the transverse and longitudinal configurations, as seen from the colour changes between 0 and 0.1 T. This indicates a stress-induced reorientation of the **Q**-vector of the helix from the weakly locked <111> axes to the stress axis. Regardless of whether the configuration is transverse or longitudinal, a uniaxial stress causes a slight decrease in *T*_c_ at 0 T. This can be attributed to the effect of volume contraction introduced by the compressive uniaxial stress, which is similar to a hydrostatic pressure effect, as confirmed by Ritz *et al*.^42^.

### Stress-driven topological phase transition

**Figure 4:**
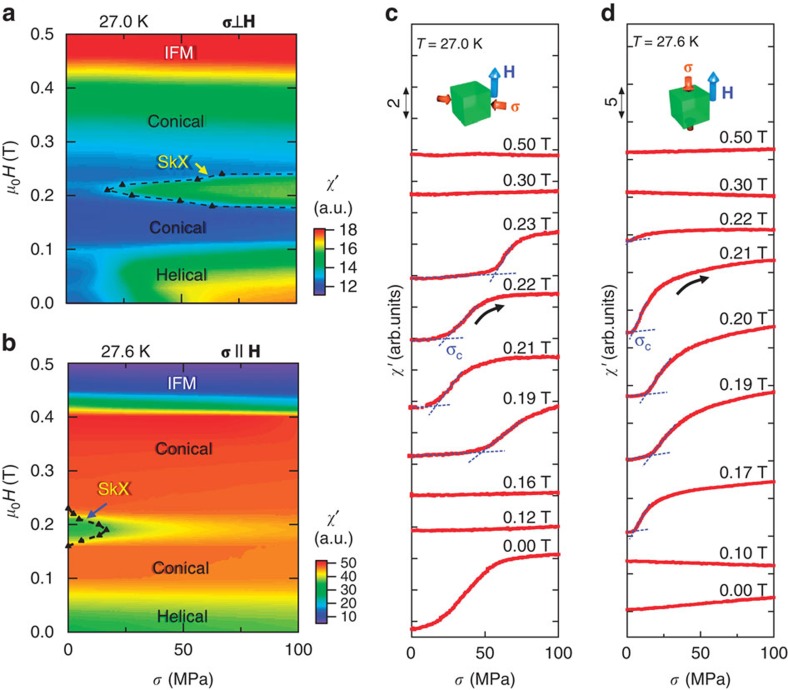
Uniaxial stress-driven topological phase transition in MnSi. (**a**,**b**) Magnetic phase diagrams in the *σ*–*H* plane under (**a**) transverse and (**b**) longitudinal uniaxial stresses. The colour maps of *χ′* were obtained from a series of increasing σ scans at 27.0 and 27.6 K, respectively. A transverse stress creates the SkX phase, whereas an existing SkX phase is collapsed by a longitudinal stress. (**c**,**d**) Corresponding *χ′* curves as functions of the (**c**) transverse and (**d**) longitudinal stresses. The large nonlinear responses indicate a stress-triggered topological phase transition at some critical stress (σ_*c*_) from the conical phase to the SkX phase in the transverse configuration, and vice versa in the longitudinal configuration. The critical stresses are plotted by (black) triangles in (**a**,**b**). Another large change in *χ′* below 0.10 T, as shown in (**a**,**b**) is associated with the reorientation of the Q-vectors towards the compressive stress axis.

The central results of the present paper are presented in Fig. 4, revealing a stress-triggered topological phase transition. Here, we continuously applied and released stresses between 0 and 100 MPa at fixed temperatures and magnetic fields. Shown in Fig. 4a is a colour map of *χ′* in the *σ*–*H* plane at 27.0 K for the transverse configuration. This figure clearly reveals the emergence of the SkX phase in the background of the conical phase at several tens of MPa. The stress-mediated topological phase transition is displayed as susceptibility stress curves in Fig. 4c. The large nonlinear responses correspond to a conical-to-SkX phase transition at a critical stress *σ*_c_. Incidentally, another large change in *χ′* at 0.00 T corresponds to the reorientation of the **Q**-vector in the helical phase towards the **σ** axis. Fig. 4b represents the *σ*–*H* phase diagram at 27.6 K for the longitudinal stress condition. The SkX phase at 0 MPa rapidly shrinks and collapses, transforming into the conical phase. The corresponding *σ*–*χ′* curves under magnetic fields between 0.17 and 0.22 T (Fig. 4d) display the topological transition from the SkX phase to the conical phase. The other magnetic phases, however, exhibit little response to longitudinal uniaxial stresses.

**Figure 5:**
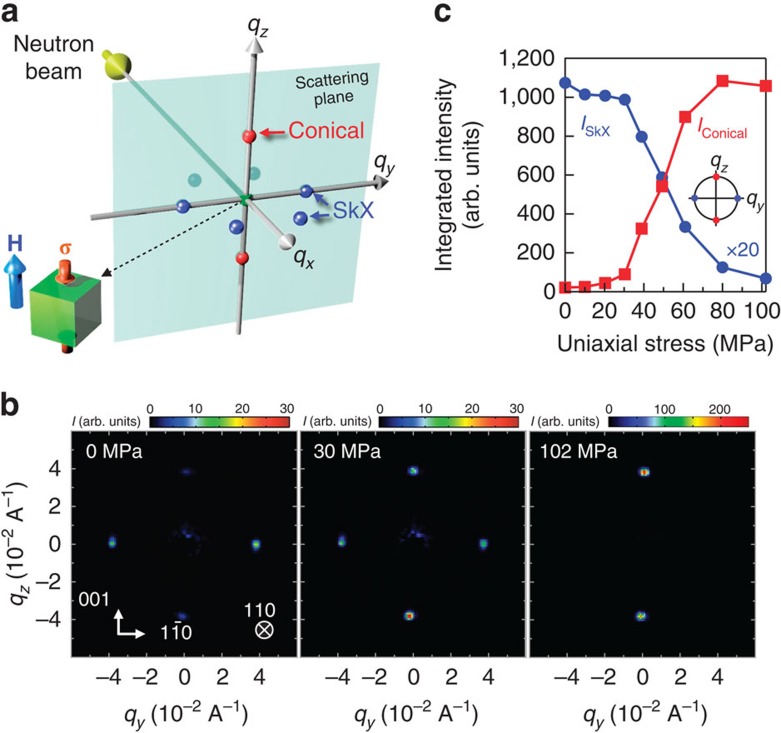
Small-angle neutron scattering (SANS) under various uniaxial stresses. (**a**) Experimental configuration for an *in situ* SANS measurement under tunable uniaxial stress. Here, **σ** and **H** were applied along the 001 direction, and the incident neutron beam was along the 110 direction. The blue and red spheres represent the positions of magnetic reflections in reciprocal space for the SkX and conical phases, respectively, and Bragg condition is satisfied when these are located in the scattering (*q*_*y*_–*q*_*z*_) plane. (**b**) Stress-dependent SANS patterns of MnSi at 27.7 K in a magnetic field of 0.19 T. With increasing stress, the major intensities shift from the ±*q*_*y*_ positions to the ±*q*_*z*_ positions indicating a stress-induced phase transition from the SkX phase to the conical phase. (**c**) Uniaxial stress dependence of the integrated intensities of the Bragg peaks for the SkX and conical phases. The relatively weak integrated intensity of the SkX phase is attributed to the existence of SkX domains, in which one of the triple Q-vectors is locked in *q*_*x*_ or *q*_*y*_ direction. The faint peaks along the *q*_*z*_ direction at 0 MPa are due to the residual conical phase.

To directly confirm the stress-triggered phase transition, we performed a small-angle neutron scattering (SANS) measurement under tunable uniaxial stress (Fig. 5a). In the same manner as for the acquisition of Fig. 4d, a longitudinal stress was gradually applied at 27.7 K under a magnetic field of 0.19 T. Because the magnetic field was applied along the 001 axis, six-fold Bragg peaks corresponding to the SkX phase appear on the *q*_*x*_–*q*_*y*_ plane. We observed two of these six magnetic reflections when the 110 direction was perpendicular to the incident beam (*q*_*x*_) and another two on the rotation of the sample by +*π*/3 about the 001 axis. These observations are consistent with a previous study reporting a weak **Q** anisotropy along the 110 direction^1^. The Bragg reflections in the conical phase, however, appeared along the 001 axis (*||* **H**). Figure 5b shows a change in the SANS pattern with an increasing longitudinal stress, in which major intensities shift from the *q*_*y*_ direction to the *q*_*z*_ direction. Figure 5c displays the stress-dependent integrated intensities of these Bragg peaks for the SkX and conical phases. The SANS results provide unambiguous evidence of the stress-driven SkX-to-conical phase transition, consistent with the *χ′* measurements.

### Phenomenological interpretation

Our study quantitatively revealed a marked effect of mechanical stress on the SkX phase in intrinsic bulk crystal. The critical stress required to create and annihilate the SkX phase near the phase boundary is several tens of MPa. The corresponding strain is estimated to be of the order of 10^−4^, assuming *C*_11_ ∼300 GPa (refs 43, 44). Previous theoretical studies have suggested that the application of uniaxial strain is an effective means controlling the stability of the SkX phase through modulation of the magnetic anisotropy^45^. Karhu *et al*.^46^ experimentally investigated such a scenario by using epitaxial MnSi films, in which several anisotropic strains were introduced by varying the thickness of the films. Recently, Wilson *et al*.^47^ also revealed that an in-plane tensile epitaxial strain on a MnSi film destabilizes the SkX phase and causes it to transform into the conical phase, consistent with our results. The epitaxial strain in this case is one or two orders magnitude larger than the strain whose effect we have investigated in this study.

Here, we discuss the strain-induced magnetic anisotropy based on the Ginzburg-Landau phenomenology. The magnetoelastic contribution (*F*_me_) to the free energy can be represented in terms of the spin density, **S**={*S*_*x*_, *S*_*y*_, *S*_*z*_}, the uniform magnetization *m*, and the strain tensor *e*_*αβ*_ (*α*, *β*=*x*, *y*, *z*) as follows^48^:


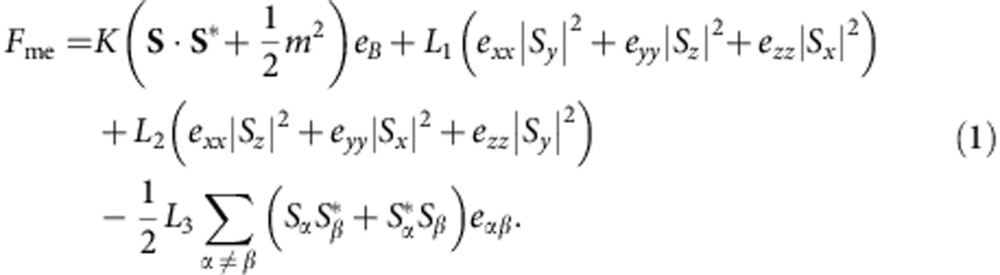


The coefficients *K* and *L*_*i*_ (*i*=1, 2, 3) originate from exchange coupling and spin-orbit coupling, respectively. The latter three terms give the magnetic anisotropy, whereas the first term gives the isotropic contribution, which is important when a hydrostatic pressure (*e*_*B*_=*e*_*xx*_+*e*_*yy*_+*e*_*zz*_) is applied^42^. Here, we consider a uniaxial stress applied along the *z* axis (that is, *σ*_*zz*_≠0, *σ*_*xx*_=*σ*_*yy*_=0). Then the energy contributing to the magnetic anisotropy *E*_*A*_ can be written as


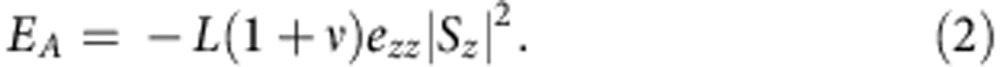


Here, we set *L*_1_=*L*_2_=*L* for simplicity, and neglect the isotropic term (|*S*|^2^=|*S*_*x*_|^2^+|*S*_*y*_|^2^+|*S*_*y*_|^2^); *v* represents Poisson's ratio (∼0.28 for MnSi^43^). Equation (2) is equivalent to that given in ref. 45, if *L*(1+*v*)*e*_*zz*_ is considered as an effective magnetic anisotropy constant *K*. Because the coefficient *L* is positive for MnSi^44^,^48^, a compressive strain (*e*_*zz*_<0) induces an easy-plane anisotropy perpendicular to the stress axis. As a result, the **Q**-vector of the proper helix preferentially aligns along the compressive stress direction. In other words, the conical phase (**Q** || **H**) should be stabilized by a longitudinal stress, whereas the SkX phase (**Q** ⊥ **H**) should be stabilized by a transverse stress (Fig. 1b,c). This explains the creation and annihilation of the SkX by transverse and longitudinal stresses, respectively.

Recently, Shibata *et al*.^49^ reported that strain also modulates the Dzyaloshinskii-Moriya interaction (DMI), inducing large-shape deformations of skyrmions in FeGe. Theoretical calculations^50^ have revealed that a uniaxial strain caused the DMI to be large along the compressive axis. This effectively induces an easy-plane anisotropy, similar to the effect of stress-induced magnetic anisotropy. Therefore, modulation of the DMI may also provide a qualitative explanation of the results of this study. However, a preliminary SANS measurement of MnSi suggests that the magnetic modulation period remains unchanged under the application of a uniaxial stress^42^. Furthermore, our SANS measurement presented in Fig. 5b reveals that the magnetic modulation period in the SkX phase remains almost unchanged on the application of a uniaxial stress; the change is <0.5%. These findings suggest that the effect of strain on the DMI is less relevant in the case of MnSi. Therefore, we interpret the effect of a uniaxial stress on the SkX phase stability in terms of a stress-induced magnetic anisotropy.

## Discussion

Advances in recent nanofabrication technologies and nanoelectromechanical devices have made it possible to utilize simple mechanical forces, instead of the conventional electrical or magnetic biases, to write information bits on a minute scale^51^,^52^. Nanoscopic mechanical imprinting has now been developed using a cantilever-based configuration^51^, which simultaneously satisfies the requirements for high densities of up to 1 Tbit in^−2^ and high speeds of up to Gbit s^−1^ in a scaled-up device called Millipede memory^52^. The concept of mechanically recordable memory, therefore, can provide an alternative architecture to various types of magnetically or electrically operated devices. Here, we propose that magnetic skyrmions can be a suitable medium for mechanical imprinting on the nanometric scale, providing the basis for a technology that can be operated at an extremely low energy cost.

The small critical stress of several tens of MPa that is needed to create/annihilate the SkX phase opens the possibility of the selective erasing of single skyrmions by simply pushing the tip of a cantilever in scanning probe microscopy (SPM). This scenario corresponds to the longitudinal configuration. By using a conventional sharp tip with a radius of 10 nm, one can introduce highly localized deformations comparable to the size of a single skyrmion in MnSi (20 nm). By approximating the contact area as *πr*^2^ (ref. 51, where *r* is the tip radius), one finds that 318 MPa can be achieved with a moderate force of 100 nN. This exceeds the threshold stress for skyrmion annihilation. In fact, nanometric ferroelectric polarization writing via SPM in a BaTiO_3_ thin film has been demonstrated to require a contact force of 1,500 nN, with a corresponding stress as large as 5 GPa (ref. 51). Therefore, although the behaviour may not be simple as that demonstrated here because of strain gradient induced by such a local force, single skyrmion deletion via SPM is plausible.

On another front, the mechanical energy needed to erase single skyrmions is estimated to be surprisingly low. This stems from the scalability of the phenomenon; the mechanical energy per bit greatly decreases with the reduction of the bit size down to the size of skyrmions. The energy cost for erasing a skyrmion can be expressed as 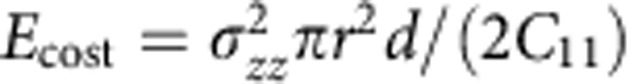, where *σ*_*zz*_ is the stress, *C*_11_ is the elastic constant, *πr*^2^ is the deformation area and *d* is the deformation depth. Here, *r* and *d* are assumed to be the tip radius and the thickness of the film, respectively. Supposing that *σ*_*zz*_=100 MPa, *C*_11_∼300 GPa (refs 43, 44), *r*=10 nm and *d*=10 nm, one can obtain *E*_cost_=5 × 10^−20^ J per single skyrmion. Although this is a rough estimate, the mechanical energy required is far smaller than the energy cost of 9 × 10^−14^ J incurred for writing to state-of-the-art spin-transfer-torque magnetic random access memory^53^ or that of 2.25 × 10^−17^ J for current-induced single skyrmion creation as calculated in ref. 32.

In conclusion, we demonstrated mechanical control of the SkX phase in a bulk MnSi single crystal, which can be achieved with a small critical stress and a low energy cost. This offers a new methodology for manipulating single skyrmions based on the conventional SPM technique. As discussed from a phenomenological perspective, the proposed mechanical approach is applicable in a broad range of skyrmionic systems with magnetoelastic coupling. By combining simple mechanical imprinting with other approaches such as a racetrack-type architecture, one may achieve novel memory or storage functions using skyrmions.

## Methods

### Sample preparation

Single crystals of MnSi were grown using the Czochralski method. We prepared two types of rectangular samples with dimensions of 4.4 mm × 2.3 mm × 2.4 mm and 3.2 mm × 1.0 mm × 1.0 mm for *χ′* measurements under longitudinal and transverse uniaxial stresses, respectively. Compressive stress was applied along the longest axes, which corresponded to 001 and 110, respectively. The external DC magnetic field was applied along the 001 direction, which was the longer (shorter) direction of the sample in the longitudinal (transverse) configuration. Therefore, the demagnetizing field was larger in the transverse configuration than in the longitudinal one. We also prepared a sample with dimensions of 3.0 mm × 2.5 mm × 2.0 mm for a measurement as shown in Fig. 3g–i, in which **H** and **σ** were applied along 111 direction. This direction corresponded to the longest axis of the sample. The surfaces and edges were carefully polished and prepared to be parallel to prohibit breaking from the edge and to introduce homogeneous compressive stress.

### *χ′* measurements under continuously variable uniaxial stress

We inserted a special uniaxial stress apparatus^39^ into a superconducting magnet of a Quantum Design physical property measurement system. The uniaxial stress was externally controlled by a combination of a micrometre and a SiCr spring coil attached to the top of the probe, and the applied force was measured by a load cell. The mechanical stress was arbitrarily controlled simply by rotating the micrometre. The spring coil ensured that the stress scan was moderate and stable. This external tunability is a notable feature that cannot be achieved using a conventional clamp-cell-type pressure apparatus. A longitudinal uniaxial stress was introduced by means of two ZrO_2_ pistons sandwiching the sample. A transverse stress was introduced by means of several ZrO_2_ pistons that converted longitudinal forces into lateral ones. Corresponding to these two configurations, the longitudinal and transverse AC magnetic susceptibilities were measured through excitation and pick-up coils covering the sample. The frequencies were 361.3 Hz for the longitudinal susceptibility and 420.9 Hz for the transverse susceptibility. We checked that the magnetic field dependence of *χ′* at 0 MPa was reproducible before and after the application of 200 MPa.

### SANS experiment

A stress-induced SkX-to-conical phase transition was directly confirmed using a time-of-flight-type SANS and wide-angle neutron scattering instrument, TAIKAN (S. Takata *et al*., unpublished observations), constructed at the BL15 of the Materials and Life Science Experimental Facility of Japan Proton Accelerator Research Complex (J-PARC) in Japan. The sample used in the SANS experiment had a rectangular shape with dimensions of 3.6 mm × 2.3 mm × 2.4 mm. We developed a uniaxial stress insert for the SANS experiment, which was essentially identical to that used in the measurements of the AC susceptibility under an applied longitudinal uniaxial stress, and loaded it into a vertical-field superconducting magnet. The uniaxial stress was applied to the smallest surfaces of the sample. The directions of the incident neutron beam, the crystallographic axes, the applied uniaxial stress and the magnetic field are summarized in Fig. 5a. The SANS patterns shown in Fig. 5b were obtained by subtracting the background that was measured after removing the sample from the uniaxial stress cell.

## Additional information

**How to cite this article:** Nii, Y. *et al*. Uniaxial stress control of skyrmion phase. *Nat. Commun.* 6:8539 doi: 10.1038/ncomms9539 (2015).
